# Role for Endocannabinoids in Spinal Manipulative Therapy Analgesia?

**DOI:** 10.1155/2019/2878352

**Published:** 2019-07-15

**Authors:** Stephen M. Onifer, Randall S. Sozio, Cynthia R. Long

**Affiliations:** Palmer Center for Chiropractic Research, Palmer College of Chiropractic, 741 Brady Street, Davenport, IA 52803-5214, USA

## Abstract

Chronic pain is quite prevalent and causes significant disabilities and socioeconomic burdens. Spinal manipulative therapy and other manipulative therapies are used to manage chronic pain. There is a critical knowledge gap about mechanisms and sites of action in spinal manipulative therapy pain relief, especially the short-term analgesia that occurs following a treatment. Endocannabinoids are an activity-dependent neurotransmitter system that acts as a short-term synaptic circuit breaker. This review describes both clinical research and basic research evidence suggesting that endocannabinoids contribute to short-term manipulative therapy analgesia. Determining endocannabinoids involvement in spinal manipulative therapy will improve its clinical efficacy when results from basic science and clinical research are translated.

## 1. Introduction

Nonpharmacologic manipulative therapies, including spinal manipulative therapy, are used to manage chronic pain [1]. Indeed, the American College of Physicians Clinical Practice Guideline recommends spinal manipulative therapy for highly prevalent chronic low back pain [2]. Despite this complementary and integrative health mind and body intervention's extensive use by doctors of chiropractic, osteopathic physicians, and physical therapists as well as its cost-effectiveness and safety [3], there is a critical knowledge gap about mechanisms and sites of action in pain relief, especially the short-term analgesia that occurs following a treatment [4]. Filling this knowledge gap will improve the clinical efficacy of spinal manipulative therapy when results from basic science and clinical research are translated.

## 2. Endocannabinoids

Endogenous cannabinoids, or endocannabinoids, have been suggested to contribute to short-term pain relief through manipulative therapy [5]. Endocannabinoids are an activity-dependent neurotransmitter system that acts as a short-term synaptic circuit breaker [6]. Like delta-9-tetrahydrocannabinol, the active ingredient in chronic pain-relieving cannabis [7], endocannabinoid effects are due to activation of G protein-coupled, membrane cannabinoid receptors CB1 and CB2 [8, 9]. These are expressed by neural and immune cells throughout human and experimental animal nervous systems [8, 10–16]. The primary endogenous ligands for the cannabinoid receptors are lipophilic anandamide (AEA, N-arachidonoylethanolamine) and 2 arachidonoylglycerol (2-AG) [17, 18]. They are formed on demand in response to increased intracellular calcium levels and are quickly degraded [6]. AEA is catalyzed from* N*-acyl-phosphatidylethanolamine (NAPE) by NAPE-specific phospholipase D and hydrolyzed by fatty acid amide hydrolase (FAAH) into arachidonic acid and ethanolamine [19, 20]. 2-AG is catalyzed from diacylglycerol (DAG) by DAG lipase *α* or *β* and hydrolyzed by monoacylglycerol lipase (MAGL) into arachidonic acid and glycerol [21, 22]. Both AEA and 2-AG are found throughout the nervous systems of humans and experimental animals [23–25].

## 3. Endocannabinoids and Manipulative Therapy: Human

Results from 2 clinical research studies support endocannabinoids involvement in short-term manipulative therapy analgesia. In one dual blind, randomized controlled trial involving male and female asymptomatic participants, McPartland and colleagues measured serum endocannabinoids levels before and after 20 minutes of an osteopathic manipulative therapy treatment or a sham manipulative treatment [26]. Osteopathic manipulative therapy included myofascial release, muscle energy, and joint articulation techniques. A high velocity, low amplitude spinal manipulative therapy technique also was included. This technique utilizes a high velocity, short duration thrust to a target joint [27]. Sham manipulative treatment was light manual contact to the heads of participants while they lay supine on a treatment table. Participants experienced cannabimimetic effects after receiving the osteopathic manipulative therapy treatment. Venous blood was collected 10 minutes before treatment and 20 minutes after treatment. Serum AEA levels increased 168% from baseline by 20 minutes after the osteopathic manipulative treatment and 17% after the sham manipulative therapy treatment. Serum 2-AG levels did not change after either treatment. The lack of changes in serum AEA and 2-AG levels following the sham manipulative treatment is in agreement with results reported from a randomized 2-session crossover study examining the short-term effects of touch massage and rest in asymptomatic participants [28].

In another prospective, blinded assessment involving male and female, asymptomatic and chronic low back pain participants, Degenhardt and colleagues measured serum AEA levels [29]. All participants had venous blood collected on 3 consecutive days. A 20-25 minute osteopathic manipulative therapy treatment was performed on day 4. Osteopathic manipulative therapy treatment techniques were soft tissue technique, muscle energy, articulatory treatment system, and strain-counterstrain. Blood was collected 30 minutes and 24 hours after the treatment. Averaged days 1-3 baseline serum AEA levels did not differ between groups. Chronic low back pain participant AEA levels did not change from baseline to 30 minutes or 24 hours after treatment. Median decreases from baseline of 34% and 42% in asymptomatic participant AEA levels occurred 30 minutes and 24 hours, respectively, after treatment. Interestingly, the decreased AEA levels seen 30 minutes after treatment contrast with increased AEA levels observed in asymptomatic participants 20 minutes after an osteopathic manipulation therapy treatment that included a high velocity, low amplitude spinal manipulative therapy technique [26].

## 4. Endocannabinoids and Manipulative Therapy: Experimental Animals

Results from a basic science research study also support endocannabinoids contributing to manipulative therapy analgesia. Martins and colleagues used an adult male mouse model of postoperative pain in which a small incision made to a hindpaw plantar surface produces mechanical hypersensitivity [30]. Inhibiting FAAH and MAGL, the catabolic enzymes of AEA and 2-AG, is a therapeutic strategy being investigated to modulate endocannabinoids for pain relief [31]. In one experiment of this study, various doses of URB937, a peripherally restricted inhibitor of FAAH [32], or JZL184, a brain permeant inhibitor of MAGL [33], were injected intraperitoneally 1 day after surgery. Hindpaw mechanical hypersensitivity was reduced by URB937 for 1-4 hours and by JZL184 at 2 hours. Subsequently, doses of URB937 or JZL184 that did not alter mechanical hypersensitivity were injected intraperitoneally 1 day after surgery. Ipsilesional ankle joint mobilization was administered 90 minutes later under isoflurane anesthesia for 9 minutes. Mechanical hypersensitivity was reduced for 30 minutes by the manipulative therapy technique. Hypersensitivity reduction was extended for 1 hour by URB937 and for 1.5 hours by JZL184. In another experiment, AM281 or AM630, selective CB1 [34] or CB2 [35] receptors inverse agonists, respectively, were injected intraperitoneally 1 day after surgery. Ipsilesional ankle joint mobilization was administered 20 minutes later under isoflurane anesthesia for 9 minutes. Reduced mechanical hypersensitivity occurring at 30 minutes following ankle joint mobilization was prevented by AM281 and by AM630. Intrathecal injection of AM281, but not AM630, and ipsilesional hindpaw injection of AM630, but not AM281, 15 minutes before ankle joint mobilization also prevented the reduction in mechanical hypersensitivity. These results indicate both central and peripheral endocannabinoids contribute to ankle joint mobilization analgesia.

## 5. Endocannabinoids and Spinal Manipulative Therapy: Future Directions

Collectively, the clinical and basic science research results described above suggest that manipulative therapy raises endocannabinoid levels. The debilitating burdens of chronic low back pain are significantly increased by a neuropathic component [36]. Pharmacological agents are the mainstay for managing neuropathic pain; however, they pose the risk of adverse effects and provide partial efficacy [37]. We developed a basic science research approach to study neuropathic pain mechanisms altered by spinal manipulative therapy [38]. A treatment of our simulation of the low velocity, variable amplitude spinal manipulative therapy technique [39] reduced hindpaw mechanical hypersensitivity during 25 minutes in adult rats that 15-18 days earlier underwent surgery for the spared nerve injury (SNI) model of peripheral neuropathic pain. Since endocannabinoids are formed on demand and are quickly degraded [6], we hypothesize that they contribute to this analgesic effect.

We have begun addressing this hypothesis with a preliminary experiment designed to obtain evidence for endocannabinoid analgesia in the SNI model. We chose FAAH as the treatment target. We used URB597 because it is a brain permeant inhibitor of FAAH that when administered intraperitoneally increases rat brain AEA levels [40]. We also used AM281 because AEA is an endogenous ligand for the rat brain CB1 receptor [17]. All methods were approved by the Palmer College of Chiropractic Institutional Animal Care and Use Committee. Using previously described methods [38], adult male Sprague Dawley rats underwent SNI surgery and were tested 15-18 days later for baseline mechanical sensitivity. Rats then were randomly assigned to 4 groups (n = 4 each group) and 2 intraperitoneal injections (1ml/kg each injection) were administered 1 minute apart to each isoflurane-anesthetized rat [38] of either freshly prepared: (1) vehicle (dimethyl sulfoxide [Tocris, R&D Systems, Inc., Minneapolis, MN]: ALKAMULS® EL 620 [Solvay USA Inc., Princeton, NJ]: 0.9% sodium chloride [Baxter Healthcare Corp., Deerfield, IL] in a volume ratio of 2:2:6) and then vehicle (Vehicle group), (2) AM281 (0.05mg/kg; Tocris, R&D Systems, Inc.) in vehicle and then vehicle (AM281 group), (3) vehicle and then URB597 (5mg/kg; Tocris, R&D Systems, Inc.) in vehicle (URB597 group), or (4) AM281 (0.05mg/kg) in vehicle and then URB597 (5mg/kg) in vehicle (AM281 + URB597 group). Mechanical sensitivity testing was repeated 15, 25, 40, 55, 70, and 90 minutes after the second intraperitoneal injection.

**Figure 1:**
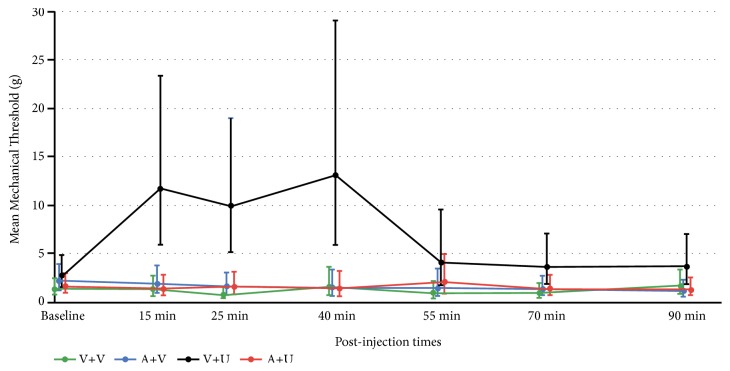
Mean mechanical thresholds (grams) on 15-18 days following spared nerve injury (Baseline) and at 15-90 minutes (min) after intraperitoneal injections of vehicle and then vehicle (V + V), AM281 and then vehicle (A + V), vehicle and then URB597 (V + U), or AM281 and then URB597 (A + U). Data are presented as means and 95% confidence intervals from the linear mixed-effects model. Vehicle and then URB597 significantly increased mean mechanical thresholds compared to the Vehicle group at the 15 (P < 0.001), 25 (P < 0.001), 40 (P = 0.002), 55 (P = 0.02), and 70 (P = 0.01) min time-points, but not at the 90 min time-point (P = 0.11). Vehicle and then URB597 significantly increased mean mechanical thresholds compared to the AM281 and then vehicle group at the 15 (P = 0.002), 25 (P = 0.001), 40 (P = 0.001), 70 (P = 0.05), and 90 (P = 0.02) min time-points, but not at the 55 min time-point (P = 0.09). Vehicle and then URB597 significantly increased mean mechanical thresholds compared to the AM281 and then URB597 group at the 15 (P = 0.001), 25 (P = 0.001), 40 (P = 0.001), and 90 (P = 0.03) min time-points, but not at the 55 (P = 0.25) or 70 (P = 0.06) min time-points.

Mean mechanical thresholds (grams) before SNI did not differ between the groups (F_3,12_=0.92,* P* = 0.46; Vehicle mean: 11.55; AM281: 15.00; URB597: 13.89; AM281 + URB597: 12.85). The group x time interaction in the mixed-effects model comparing groups across the post-SNI time-points was statistically significant (F_18,12_=8.03,* P* < 0.001). SNI decreased baseline mean mechanical thresholds 15–18 days later to a similar extent across all groups and maintained those mechanical thresholds for the Vehicle, AM281, and AM281 + URB597 groups (Figure 1). In contrast, intraperitoneal injections of vehicle and then URB597 consistently increased mean mechanical thresholds at the 15, 25, and 40 minute time-points and then decreased to a mean higher than those of the other 3 groups at the 55–90 minute time-points. The results indicate that endocannabinoids are a treatment target for peripheral neuropathic pain produced by SNI.

## 6. Conclusion

There is a critical knowledge gap about mechanisms and sites of action in spinal manipulative therapy analgesia that when filled will improve clinical efficacy. Having demonstrated that endocannabinoids are a treatment target in the SNI model of peripheral neuropathic pain, we next will use behavioral pharmacology approaches from the ankle joint mobilization study described above [30] to determine whether endocannabinoids contribute to the analgesic effect of our simulation of the low velocity, variable amplitude spinal manipulative therapy technique.
